# Vitality from Experiences in Nature and Contact with Animals—A Way to Develop Joint Attention and Social Engagement in Children with Autism?

**DOI:** 10.3390/ijerph16234673

**Published:** 2019-11-23

**Authors:** Kristina Byström, Patrik Grahn, Caroline Hägerhäll

**Affiliations:** 1 Department of Work Science, Business Economics and Environmental Psychology, Swedish University of Agricultural Sciences, SE-230 53 Alnarp, Sweden; patrik.grahn@slu.se (P.G.); Caroline.Hagerhall@slu.se (C.H.); 2 Region Västra Götaland, Habilitation & Health, Children and Youth Habilitation, 541 50 Skövde, Sweden

**Keywords:** autism, child development, treatment, mentalization, animal-assisted therapy, nature-based intervention

## Abstract

Animals are increasingly included in treatment for children with autism, and research has shown positive effects, such as increased social initiatives, decreased typical autistic behaviors, and decreased stress. However, there are still knowledge gaps, for example, on underlying mechanisms and effects from longer treatment duration. The purpose of this study is to contribute to these gaps and ask questions about the ways in which animals and nature can improve conditions for psychological development through support from therapists. The method is based on grounded theory. Data comes from a treatment model (duration 1½ years, a total of nine children), from environmental psychology and developmental psychology, both typical and atypical as in autism. The results consist of three key categories; reduce stress and instill calm, arouse curiosity and interest, and attract attention spontaneously. These three key categories are related to an underlying core variable, vitality forms, which was described by Daniel Stern and, according to him, is important in forming overall experiences. The starting point is the brain’s way of encoding many internal and external events based on movement perception. Here it is argued that the vitality forms from nature and animals are particularly favorable for effecting development-promoting interactions with a therapist.

## 1. Introduction

Nature and animals are increasingly used in treatment and educational contexts [1]. Natural environments have been shown to reduce stress [2,3], increase the ability to focus attention [4,5], increase curiosity, motivation and commitment to learning situations [6]; and increase opportunities for physical and emotional activity through playful activities in nature [7,8,9]. Systematic reviews provide a basis of knowledge for the statement that introducing animals and nature into treatment and pedagogy has demonstrable effects [10,11,12,13,14]. For the clinical group of children with autism, several studies have reported an increase in social initiatives, a decrease in typical autistic behaviors, a reduction in stress, and a lower level of anxiety during therapy sessions when the children were accompanied by a dog or a guinea pig [15,16,17,18,19]. Improvements are also reported in behavior, social interaction, and communication after treatment with equine assisted therapy [20,21]. To the best of our knowledge, there are no studies where therapists, with the help of nature and animals, provide more long-term psychological development support to children with autism. This study is based on empirics from a treatment model with this purpose, and therefore contributes to knowledge in the field.

### 1.1. Autism

Autism involves limitations on mutual social interaction and communication as well as limited variation in behaviors, interests and play [22]. Both verbal and nonverbal communication are affected. The prevalence is approximately 1% of the population [23,24]. The condition is complex and the group of children with autism is a heterogeneous group [25]. Individuals with autism often display comorbidity with other disorders such as hearing and visual impairment, epilepsy and attention deficit hyperactivity disorder (ADHD) [26,27]. Of all children and adolescents with autism, 20%–40% also have an intellectual disability [28,29]. More than 90% of children exhibit aberrant sensory reactions [30]. The DSM-5 criteria [22] now include hyper- or hypo-reactivity in sensory stimulation or special interest in sensory aspects of the environment. For example, children with autism may react strongly to specific sounds, be visually fascinated by light or movement, etc. Problems with emotional regulation are common, as well as high anxiety levels compared to many other clinical and non-clinical groups [31,32]. Another difficulty is sense of time [33,34], which many children with autism have difficulty developing.

As yet, research has not been able to agree on any specific theory that explains the underlying mechanisms of autism [35], but over the past 20–30 years, research has highlighted primarily the following three areas of cognition that works differently; mentalization, that is, the ability to attribute oneself and other persons thoughts, feelings, desires and intentions and the understanding that these states affect people’s actions [36], executive function, that is, the ability to plan and perform complex actions [37,38], as well as what is called central coherence, which means the ability to link information to wholes rather than just seeing details [39,40]. 

Regarding the development of mentalization, in young children with autism, it is severely delayed, deficient, or alternatively not at all occurring [41]. Also, abilities that in children with typical development are considered to be linked to early mentalization development such as the ability to share attention, mutual communication, and pretense [41,42,43], are limited in children with autism [44]. Promoting these abilities in treatment is commonplace today and is considered to create the conditions for children with autism to develop their social ability, their language [45,46,47,48], their cognition, and mentalizing ability [49]. Studies have shown that children with autism who pass tests for mentalization (a more advanced form, developed at about age four in children with typical development) solve the tasks linguistically and not through a more intuitive social insight ability [50]. According to Hobson [49], to achieve a more intuitive ability it would be required to experience a more direct way of becoming emotionally involved with others and not only through a process of a conclusion. In his view, children with autism have difficulty with this. In other words, the gradual development of mentalization through experiences of natural social interaction episodes that children with typical development undergo in a safe and stimulating environment, children with autism have significantly poorer conditions to reach.

### 1.2. Treatment of Autism

Interest in developing treatment for children with autism has increased in recent decades. Today, there are a variety of models to support the psychological development of young children with autism [51,52,53,54,55,56] and with varying results [51]. Many of the treatment models provide support in a very structured way, but some have a more naturalistic approach and follow the children’s own initiative to a greater extent [57]. No treatment has yet been found to suit all children with autism [55]. Research conducted to date shows that the Intensive Behavioral Therapy (IBT) model based on learning theories [58] has the best results, especially for children with intellectual abilities within the normal range [59,60]. The model is a type of educational intervention, which is based on the fact that preschool children with autism practice a range of different skills in small steps during 25–30 hours a week. Intellectual impairment in autism means a reduced opportunity to learn through cognitive learning [61]. A nature and animal-based treatment method aimed at children with autism, which we use in this study, is KOMSI ® (COMmunication and Social Interaction), (see Materials and Methods below). 

According to Rutter [62], the primary goal of treatment for autism should be to support normal development. This is not easy because the cognitive difficulties that are believed to be behind many of the behavioral problems along with the neurological immaturity, hinder the early experience-based development that children with typical development undergo in relationships and interactions with other people in a safe and stimulating environment [62,63,64,65,66]. What is a stimulating environment for children with typical development can be over- or under-stimulating for a child with autism [62]. The social and communicative difficulties together with hyper- or hypo-sensitivity make it difficult for the daily experiences of interaction that the environment offers to be utilized in a development-promoting way. Charman [67] argues that the lack of developmental promotion experiences in itself negatively affects the following psychological development. Creating improved conditions for this so-called experience-based development is therefore very important in order to reduce the negative consequences in the long run. 

### 1.3. Aim

The aim of this article is to look for a theoretical model that deepens the understanding of the mechanisms underlying the positive effects of involving nature and animals in a developmentally supportive treatment for children with autism. Using such a theoretical model, existing treatment methods involving animals and nature could be further developed or new methods could be developed.

## 2. Materials and Methods 

### 2.1. Material

The material comes from the first author’s clinical experience, most of which comes from her practical experience from the treatment in the project KOMSI® which includes observations and process notes. It is supplemented with scientific literature, discussions with colleagues, seminars, etc. according to “grounded theory (GT)”, see below under “Analysis”.

#### 2.1.1. The Treatment Model KOMSI ®

KOMSI ® is about a 1½ year long, group based, nature- and animal-based interaction and communication treatment, aimed at children with disabilities, mainly autism, both with and without intellectual disability, and with a mental age range of 4–6 years, and speech development at a quite early stage. The model has been tested on two separate occasions [68,69].

The treatment environment is located on a small farm with animals and with the surrounding natural environment where the children take part in horse riding, games (e.g., hide and seek, chase and run, coppers and thieves, and shipwrecking) and other related activities with therapists. The sessions are 2½ hours long and take place during school hours, once a week, about 40 occasions in total, and the group consists of 4–5 children, 6–7 therapists (i.e., psychologist, physiotherapist, occupational therapist, special pedagogue, teacher, and teacher´s aide), and a person responsible for the animals. 

#### 2.1.2. Participants in KOMSI ®

Treatment with KOMSI ® was performed in two different projects with four children in the first group and five in the second. All nine participants were recruited from two habilitation centers in medium-sized Swedish cities. All but one child had been diagnosed within the autism spectrum, all before the start of treatment, and all but one, also had an intellectual disability at a mild level. One child had an acquired brain damage and a mild intellectual disability. The procedure and assessments leading to diagnose were carried out by a psychologist and a doctor in the specialized health care. The tests used were ADI-R (Autism Diagnostic Interview-Revised) and ADOS (Autism Diagnostic Observation Schedule) [70]. The children’s ages were 6–8 years old at the start of treatment. Mental age in the first group was about 5 years old and in the second group about 4 years old, but in the last group the verbal expressive level was somewhat lower, about 3 years.

Informed consent was obtained from the children’s parents regarding photos and other empirical data included in this article. Most of the examples from the children’s communication and statements included in the paper are considered to be highly similar to the children’s actual statements. This is because the therapists stated them from memory directly after the treatment sessions. Some examples are exact, because they were recorded on film. The photos presented are taken by the first author in all except in one case, where the photographer instead was the therapist Ingrid Bertilsson.

The research has been approved by the Ethics Review Board EPN, 2007-10-23, T626-07, Ad Ö 348–01; 2009-08-17 Dnr 416–09. Animal ethics has been guided by the commonly-accepted ´3Rs´ [71]: Replacement of the horses and the dog to nonalive objects or to reduce the number of horses in the study were no alternatives. An example of refinement was applicable in the occasion where a horse was replaced, as the relation between horse and boy was considered non-optimal. 

#### 2.1.3. Data from Treatment with KOMSI ®

The data consists of process notes from the participants’ development during the treatment. Information about the context of the children’s behavior in the treatment was also written down as the therapists purposefully involved animals and nature for their interventions. These notes are based on the therapists’ shared reflections on the children’s behaviors that occurred after each treatment session. The same procedure was used related to which photos should be picked out and sent home to the participating children (second project), after each session. In this way, the data has undergone a form of validation continuously. One practitioner’s viewpoint on a child’s behavior may have been contradicted or confirmed by another practitioner’s statement and consensus has been sought to obtain the most likely understanding of the events. 

When the children’s development progress was evaluated, this was done within the group and under supervision. All therapists from the habilitation center had access to the notes during reflection time or supervision as they were collected in a special folder, one for each child. All aspects, both positive and negative about the children’s behavior and reactions, were written down, as all therapists were motivated to help the children to progress with their development during the therapy. For the therapists, the treatment was primarily a long-term treatment in the habilitation service and not a research project. Possible observational effects or Hawthorne effects mainly arise during short studies and/or where personal rewards are evident, which is not considered to be valid in this case [72,73]. 

Additional data from the treatments referred to above consist of the first author’s interviews of parents, teacher and parent surveys, tests, conversations with colleagues, and films and photographs from treatment sessions, but also from the author’s impression from conferences, participation in workshops, seminars, and trainings, as well as from her clinical work beyond KOMSI® but where also animals and nature were included. These experiences have been used as a basis for comparison with the main empirical material that is the process notes.

### 2.2. Analysis

Data have been analyzed using the qualitative method grounded theory (GT) but with elements of abduction in the analysis method. GT is a method of generating new theories through induction [74] or when one wants to develop and renew existing theories by adding new aspects [75]. GT has become an increasingly common method of studying social phenomena where theory formation is lacking [74]. In GT, both qualitative and quantitative data can be used, but with emphasis on qualitative data and in the analysis, elements of deduction can occur. According to Reichertz [76], abduction can also occur within GT, a form of conclusion where collected data is combined into new combinations and where existing theories are not sufficient to explain the patterns you see. The search for explanations can then lead to new knowledge and new discoveries.

Already the founders of the method, Glaser and Strauss [77] had different views on the method and how it should be used, so it is common today for researchers to state that they are inspired by GT, which is also done here. Glaser, for example, wants to give the researchers great freedom; that their creativity should be used as much as possible and believes that it can be done without taking away the rigor of the scientific work. According to him, the validity arises if the reader when he reads the results can recognize himself, and get a feeling that this sounds reasonable. Here, Glaser’s approach is used by reusing data derived from previously performed research projects; one unpublished paper during studies in psychology in 1998 (A literature overview about animal-assisted therapy (AAT) for children with autism and an evaluation of an animal-assisted activity (AAA) program involving children with autism and intellectual disability visiting a farm), and the two research projects of KOMSI® [68,69].

The first step in the analysis was to encode the themes of children’s development that were described in the first project with KOMSI ® [68] and in the second [69]. Episodes that were considered to contribute to children’s development (e.g., exploration, play, social interaction, and communication) were coded with so-called open codes, i.e., they have received descriptive texts linked to them. Content that was repeated was found and explored and eventually this work led to the emergence of certain main themes. In the second stage of the analysis, the outlines of each main theme were refined and examined based on how they were linked to each other, as well as the literature related to theories of children’s typical and atypical development such as in autism and to the field environmental psychology concerning nature and animals for children and adults’ development and health. Of these main themes, four key categories were now formed 1) Reduce stress, 2) Awaken curiosity and interest, 3) Use of attention spontaneously, and 4) Vitalize and give energy. It was discovered that all four themes had some overlap with each other and all four could be linked together and individually to the three theoretical directions mentioned above (children’s typical development, atypical in autism, theory from research on nature, and animal significance for human health).

In a third and final step, the fourth key category of Vitalize and give energy was transformed into a main category of the four key categories, which the other three key categories came to revolve around. This main category is referred to here as the core variable. The process of finding it came from the fact that the fourth key category was larger than the others in its abundance of thoughts and associations, and upon closer analysis it was found that it also had explanatory value for each of the other three key categories. The core variable does not give rise to a completely new theory, which is usually seen as the purpose of GT, but to an already known theory, a theory that, as far as the authors are aware, has not previously been applied to children with autism in the context of treatment with support from nature and animals besides the support from therapist. It is about Stern’s thoughts and theories on how the feeling of being alive arises in a human being, so-called vitality, and its importance for psychological development, for all our daily experiences, including those about reading and understanding other people’s mental states. The vitality forms, are a type of perception that arises from how one interprets movement, says Stern [78]. They follow our experiences by adding form and dynamics to our thoughts and feelings. Stern explains that we do not notice them because we concentrate on the content of our experiences [78]. He believes that they have an important integrative task in the mind as they fuse impressions and help us to perceive them as dynamic events in the form of holistic experiences (described in more detail in the results section). The article examines the possibility that the registration of vitality forms, here freely interpreted as movement perception, may have explanatory value for how children with autism can read meaning into what is happening in a treatment environment consisting of nature and animals.

## 3. Results

The study revealed three key categories and one core variable. The core variable is described directly in the presentation of the key categories but also in the end of them as a short summary. Since it is easy to confuse vitality forms with mental content and because the theory is not a well-known theory, the presentation of the results begins with a more general theoretical description of the concept of vitality.

### 3.1. The Concept of Vitality

#### 3.1.1. Vitality and Vitality Forms 

The aspects of the concepts of vitality and vitality forms that are addressed come from Stern’s latest and more extended version of the subject [78]. It is about aspects that specifically touch on Stern´s thinking how they arise within a human being, the importance of them in relation to mental content such as thoughts, feelings and sensations from which they are separate but still affect and are affected by [78], and finally, the importance they have for mentalizing and the connection in this regard to children with autism. 

A short quote from Stern’s book ([78], p.4), may be used here, as it well seems to capture the essence of vitality for the human experience and at the same time have relevance for the way people with autism seems to grasp the world. Their cognitive style is characterized by being focused on detail, facts, and is more derived from logic than from an integrated whole. In addition, children with autism sometimes seems fascinated not by mental content in itself, but more of the dynamics that moving things or light phenomena can create in mind.

*”Without manifestations of vitality, the world would be bereft of much of its interest, and human interactions would be digital rather than analogic, whatever that might be like”.*

#### 3.1.2. Human Experiences Based on Vitality and Forms of Vitality

Stern describes that vitality arises as a result of how the brain integrates many internal and external events (those that take place in moments of seconds) so that total experiences arise [78]. However, experiences must have their basis in some form of movement, mental or physical [78]. As movement perception is always accompanied by the four qualities, force, space, time and direction/intention, vitality is also experienced with varying degrees of dynamics. A movement, for example, occurs during a certain period and therefore a sense of time is coded. Movements also takes place spatially, which adds a dimension of space to the movement perception as well as a sense of intentionality, since movements always have a direction [78]. Stern points out that we are constantly moving in some way, e.g., through breathing, when we speak, through gestures, mimics, or gaze directions, and that every movement has their respective time contours and flows [78] and through the mirror neuron we can perceive another person’s inner state by watching how they move. Mirror neurons are visual/motor neurons in the brain that are considered to be linked to social and empathic behaviors [79]. They are activated in the same way when an act is seen to be performed, as when it is performed by the person himself [79]. In this way, the forms of vitality are important for social understanding between people.

The vitality forms are almost always intertwined with feelings and thoughts but can occur without content. An example of this according to Stern, is when infants communicate with their caregivers (part of affect attunement, the way inner states of the infants are mirrored back to them [80]). Or for a brief moment after a stimulus (for example as when looking at a picture) but before thoughts and feelings are initiated [78]. They are part of the episodic memory and a small movement can remind us of an experience we had in the past and with the help of the forms of vitality brought about, an entire memory of the event can be recalled [78]. To date there also exist some scientific support on neurological underpinnings of vitality form processing in the brain [81,82]. 

#### 3.1.3. Children with Autism and Registration of Vitality Forms in Another Person’s Expression

There is very little research on vitality forms and children with autism but in a study by Hobson and Lee [83] it was shown that children with autism could imitate another person’s action, but rarely the way the action was performed by the typically developed children in the control group. This, Stern proposes [78], could be explained by the fact that the children with autism cannot imitate the forms of vitality of the action [78]. Hobson and Lee [83] argue that children with autism have difficulty in interpersonal engagement, which is required to imitate the way an act is performed. Stern was part of a research group that conducted a similar study [84] where the ability to see similarities and differences in actions performed with the same or different form of vitality was examined in a group of adolescents with autism. The result showed that the group with autism compared to control had clear difficulties in perceiving the forms of vitality in the performance. The authors propose that these difficulties may be at the root of the social difficulties in autism which could affect the ability to be socially toned and to be able to mentalize [84]. The authors also conclude that the new insight may lead to development of clinical interventions for individuals with autism.

### 3.2. Key Category 1—Reduce Stress and Instill Calm and Peaceful State of Mind

For young children, strong negative emotions are associated with experiences of stress and they need help with regulation of affect [65,85]. Children with autism have easily aroused stress due to disabilities and more difficulty in using so-called social strategies such as turning to other people for help. Therefore, finding other complementary strategies for the group that can provide them with improved emotional stability is very important, especially when receiving treatment. According to Ulrich [86], the reason why nature is an especially salient environment for humans from the perspective of stress reduction is due to the fact that man has evolved in nature over a very long time and therefore adapted to it both physiologically and psychologically, something that has not yet happened for the urban environment. There are also explanatory models other than the so-called evolutionary as to why the natural environment can work as soothing to humans and provide help with stress reduction. One example is the Perceptual Fluency Account theory (PFA) [87,88], which, like evolutionary theories, is based on a biological mechanism. According to PFA, the explanation for nature’s positive effects on attention and stress can be found in the fact that our visual system processes the information in natural environments with greater ease than information from other environments. One factor that helps visual structures in nature be easily processed is the high degree of coherence and order created by repeating shapes that are similar to each other, something that with a mathematical concept is called fractal geometry. Stern’s theory of vitality and vitality forms can also fit well into this key category of stress reduction and provision of calmness based on all the gentle repetitive and slow movements without stressful direction/intention occurring in nature.

For children in the treatment, it seems that nature and animals have helped reduce stress in different ways, both directly and through the therapists’ interventions. An example of the latter could be when therapists use nature and animals as a distractor when contact from direct experience of the interaction with themselves, or other group members, threatens to become too intense and overburdening. The animals, together with the clarifying verbal and non-verbal emotional support from the therapists, can then be used to regulate the arousal level in the child. It seems that the children’s heightened interest in the animals makes it possible for the therapists to relatively easy catch and draw their attention from something that caused them concern, to other elements in the environment, which then reduce stress as a result. The children have also independently been seen to switch their focus of attention when they, for some reason, have not been able to, or wanted to remain in the communication with the therapists or other group members. They may have felt stressed for some reason but also tired or in need of more processing time. Often on such occasions they have looked for an animal or just walked away for a while in their own thoughts and with their eyes fixed on something in the environment. The question is whether nature and animals then functioned as a kind of safety valve against further stress to build up, which at the same time may have facilitated mental processing to be done more efficiently. Eventually, the children returned to the therapist or someone else in the group and then again often showed interest for what was going on. In other words, it has not always been easy to explain why stress, or signs of discomfort, appeared to decrease in the children as it could be many factors contributing to this. If the main reason was about fatigue or need for more time to process information, a slow wandering gaze towards any indeterminate movement from an event in nature may have resulted in the children regaining vitality through the activation of soft forms of vitality that are easy to interpret based on direction/intention. It could be from watching the movements of grazing animals or looking for a while at a tree canopy swaying softly in the wind or listening to the quiet whisper from the moving leaves of the trees. 

Another more indirect example of stress reduction is through therapists’ interventions when the animals’ behaviors were used as a kind of mirror and as starting point for conversations about conditions the children themselves did not understand so well. The children have not always shown clear stress in these situations, but more worry or confusion, and may have silently looked at something and tried to grasp the meaning of the event. An example was when a child left the group after an episode of interaction with the dog and another boy. The therapist saw that the boy looked frustrated, standing silently next to the fence outside the playground in the forest. The therapist approached him and began to gently make contact. Her hypothesis was that the boy could not handle that the other boy also interacted with the dog and therefore he withdrew. When she confirmed him with words, she chose to take the perspective of a raven (they were in the area) because she did not think he was ready or motivated to listen otherwise. She described, from the bird’s perspective, what she thought had happen, and then asked him if the raven was right. The boy answered yes, looked relieved and joined the group again. In this and similar ways, the animals were sometimes included in the therapist’s interventions. Words were used to form important linguistic links to emotional states, which seemed to increase the children’s understanding with reduced stress as a result or as here, helped to break an emotionally locked state.

Another type of event that may have increased the children´s sense of safety, was when the children watched when animals communicated and played with each other. This was a kind of experience that amused them a lot and made them laugh, and the episodes also elicited vitality in the children. The domestic animals in the farm (pigs, and chickens), along with those grazing in the pastures (cows, and horses) and those involved in the direct interaction (a horse and a dog) may, together with nature’s structures, have signaled to the children that the surroundings are a safe, interesting place to be in and a place that is easy to perceptually understand. This is what Ottosson et al. in their article on Parkinson’s disease, label a biophilic environment [89]. When the animals are calm, they convey the message that no danger prevails. It is also a great advantage that many of the animals’ behaviors could easily be linked to human behaviors and human needs such as; play, food, rest, or to show emotions in the communication with others. This has made it easier for the therapists to be able to offer the children a varied and tailored knowledge of human’s life conditions, that are believed to have broadened their perspectives and understanding of links between behaviors and the mental.

Animal behavior can often be perceived by humans as relatively easy to interpret based on intention (the horse wants to eat as it tries to snap leaves from the trees as we ride, the dog asks with a praying gaze and waving tail for a piece of food, etc.) which is probably a great advantage for children with autism who have difficulty interpreting intent behind human actions. Their mentalizing ability can thus be enhanced more directly by looking at animals’ behavior and understanding what they see. In other words, the behavior of the animals in the treatment and the therapists’ ways of helping the children with the linguistic understanding may have led to stress reduction through increased comprehension of what is happening. The children’s contact with the animals also relies heavily on the animal’s acceptance and less demanding nonverbal communication. The children with autism seem to benefit from this, as they have difficulty interpreting human communication, which almost always involves both nonverbal and verbal modes of communication, and so is much more complex. If the children also register the vitality forms when looking at the animals’ behaviors when talking to, or listening to their therapists, that could also have contributed to them getting a more coherent picture of the whole event. Especially as emotions and vitality forms mutually affect one another, according to Stern [78]. Perhaps also time registration could become more efficient by vitality form registration, and hypothetically help the children to develop their sense of time, an area that is often impaired in autism [33,34]. Perceiving movement from alive things is a very early form of perception that is already in place in very young children. One-year old children see geometric figures in experimental studies as intentional, based on how they move [90,91], and three to four-year-old children attribute desires, goals, and emotions to such geometric figures [92]. Animated perception is considered to be an early milestone in children’s development [93], that contributes in an integrative way to the social information process and social cognition of children [94]. 

When it comes to children with autism, however, there is very little research on how they perceive animated movement. Rutherford and colleagues [94] saw in their experimental study that children with autism, compared to controls, took longer times to learn to perceive animated movement based on geometric figures presented on a computer screen, but that they learned in line with the control groups. However, Klin [93], who did similar studies but without learning phase (adults with autism), found that individuals with autism performed worse than the control. Rutherford and colleagues [94] raise the notion that the neural mechanisms of perception of biological movement may be intact in children with autism but that they may need to learn to perceive them. The question is whether the children with autism can decode movements of animals automatically, if they find them interesting? If so, the function to read biological movement is stimulated. This should be an advantage, since animal movements just like human´s, are intentional in some way and can give rise to thoughts and behaviors which can promote valuable experiences for the children to develop their social understanding and mentalization further.

Another aspect of human communication with animals that may be of importance, is the use of a softer tone of voice. Research has shown that when people talk to animals, they often use similar modes of communication as when talking to young children (so-called motherese), i.e., a form of word-filled conversation, repetitive and slow, and filled with soft, calm vitality forms and facial mimicry [16]. This type of communication with more musical prosody used by the therapists occasionally when talking to the animals, seemed also to have increased the children’s interest and vitality for what was happening in the interaction. Sometimes calmness from this situation seemed to transfer even to the child’s emotional state. Some children have also used this softer more musical way of talking to the animals themselves. A boy first spoke with a softer voice directed to the farmer’s car, which he liked very much, but after a while we heard him use the same soft voice when talking to his horse. He seemed to have generalized the way of speaking from one situation to another, something that is not so common in autism. Similarly, the therapists’ more emotionally colored tone of voice when speaking, may have been transmitted to the children, suggesting that affective transmission may have occurred through mirror neurons. Such a possible transfer may also be influenced from other kinds of movement perception than from prosody, for example, from the children´s awareness of the whole therapist/animal situation, taking in also the vitality forms from the therapists’ gestures and body language. Katcher and Friedman [95] have shown in their research that conversations with animals reduce stress and blood pressure in humans, which is not the case in conversations between people. It may also apply to individuals with autism.

#### 3.2.1. Theories and Mechanisms in Relation to Calm and Peaceful State of Mind in Children

**Figure 1:**
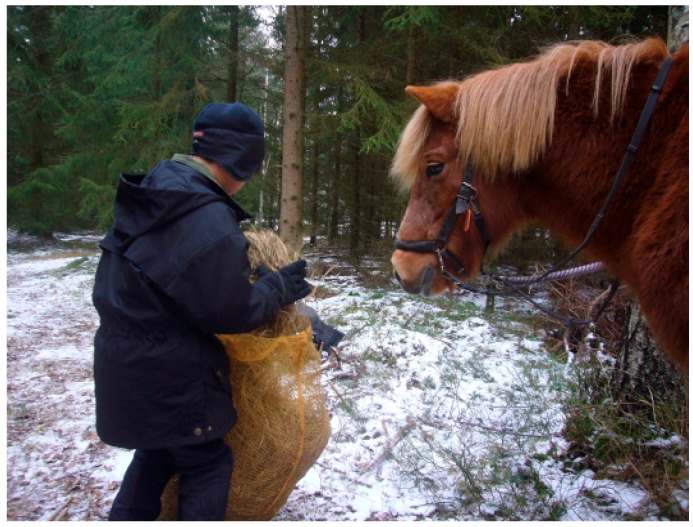
Giving the animals food is a way to show care and it is a valuable experience for children with autism as it is not easy or natural for them to get that kind of experience in relation to other people.

**Figure 2:**
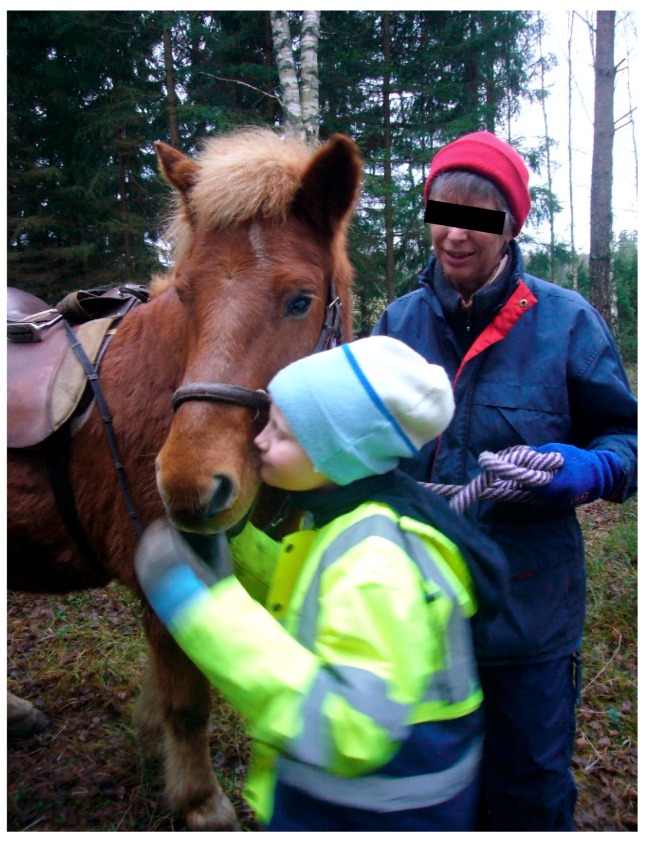
Being able to experience a good relationship with a horse in therapy can contribute in a non-threatening way to children with autism to experience a kind of fellow ship with another living being.

Oxytocin is a neuropeptide involved in the regulation of several different types of social links, both in animals and humans through calming effects [96]. One example is behaviors between children and parents such as mothers breastfeeding their young children [96], but oxytocin release also occurs from people’s physical contact with animals, especially by patting dogs [97]. It is about the hormone opening up to systems that have to do with attachment [96]. It is primarily through enjoyable tactile interaction that oxytocin is released but it can also occur through eye contact as in a single encounter with an animal, according to Beetz et al. [98]. In the treatment we see that the children often look for the animals close by and stop for a while to pat or look at them. For some children, this has been especially important. Most children though not all, have repeatedly shown the animals care in the form of talking to them in a soft voice, sometimes taking the animal’s part in conversation with someone, or showing the animals another type of care such as giving them food, see Figure 1, or patting them, see Figure 2. 

**Figure 3:**
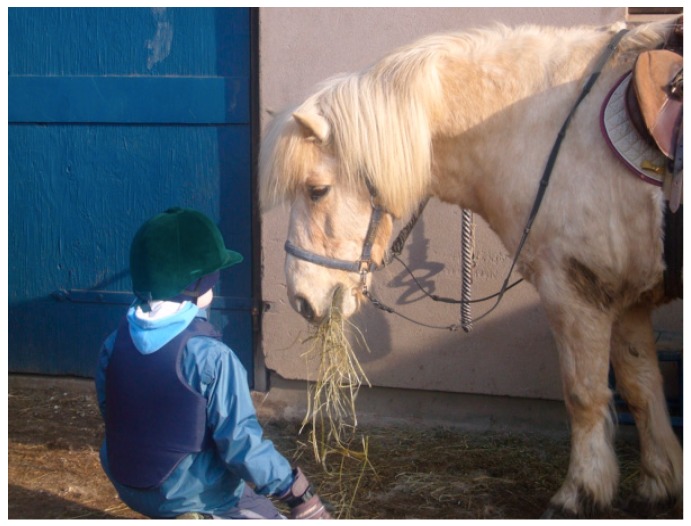
An animal is likely to offer a child with autism a calmer form of contact than is possible from people.

Beetz et al. [98], who do research on human-animal interactions, indicate that the fact that mammals and humans share behavioral systems for attachment and care and have the same biochemical hormonal basis for these behaviors, is important for these interactions between animals and humans to often work well. There are also several examples from the treatment that the children were able to respond to and were interested in episodes about proximity, contact and care, based on situations communicating with and about animals. These are assumed to be important examples of social and emotional experiences that children with autism cannot so easily gain from interaction with people. These experiences therefore help them develop trust and a sense of security, which are good conditions for developing other abilities, such as how to talk about needs and behaviors that humans can have also. According to Beetz et al. [98], activation of the oxytocin system is suggested to be one of the underlying mechanisms behind the positive effects of human–animal interactions. Stress reduction based on oxytocin effects may also be involved when children with autism interact with animals in a way they like and seem to be calmed by, see Figure 3. We have seen many examples of this effect from the treatment included here. Oxytocin could hypothetically also be released based on the theory of vitality forms, for example from soft speech, grazing cows, and from chewing animals such as sheep, cows, and horses. 

In recent years, research on oxytocin and individuals with autism, mostly adults, has increased. A review by Stavropoulos and Carver [99] describes improved performance on tests for social cognition after oxytocin intake for the group compared with control. Studies that specifically focused on oxytocin in conjunction with intervention regarding joint attention in autism, argue that the lack of social motivation in the group may impede the long-term positive effects [99]. For children with autism, it is conceivable that the motivation for shared attention with humans increases if the interaction involves animals and play in nature as those events often are perceived as positive and motivating. In the treatment that has often been the case. 

**Figure 4:**
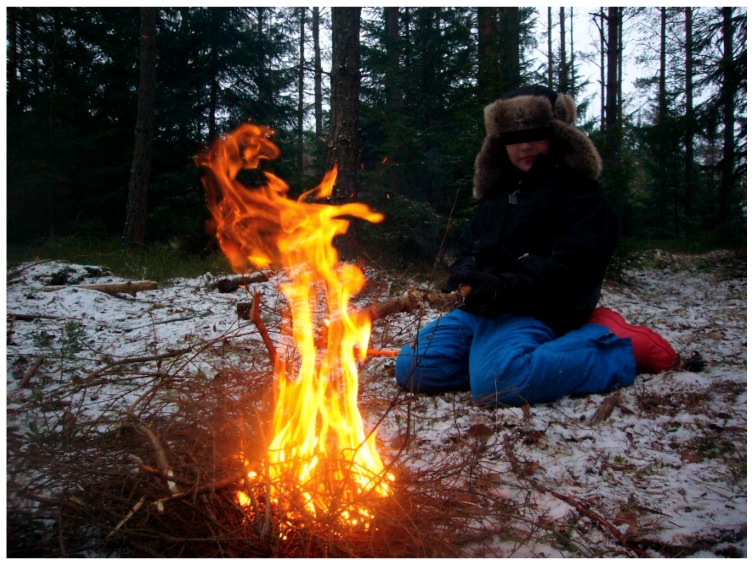
Heraclitan movements, movements that are recurrent and still remain the same, intrigues this boy. For him, the fire is very important to look at but periodically also to take more active care of.

Stress recovery theory (SRT) assumes that certain structures in nature can trigger rapid positive emotional reactions in humans, which is considered to be a result of evolution where it has been of great importance for quick recovery after stressful events [86]. Examples, according to Ulrich [86], could be structural features in nature’s formations such as symmetries, depth, and absence of threats. Further, he states that particular features of visually perceived nature can be particularly linked to feelings of security and thus restoration, such as calm and slow-moving water, or movements from a small controlled fire [100]. Here is an opportunity to bridge over to Stern’s theories of vitality based on gentle recurring movements in nature. Perhaps these kind of vitality forms can lead to a reinforced sense of security when looking at them. It is difficult to know if the children in the treatment have reached stress reduction or gained increased emotional stability from looking at that kind of movements in nature and whether registration of the vitality forms is a key mediating factor, but hypothetically, that could be the case. For example, during riding when the children often appeared relaxed and sat silent on the horse with their eyes fixed on something in the surrounding nature that moved in the manner described earlier. Another example would be when they were sitting on the ground looking into the flames of the small campfire, often with a satisfied and calm facial expression, see Figure 4.

Researchers other than Ulrich also highlighted nature’s formations as a source of stress reduction. For example, Wohlwill [101], noticed in his research that people like to look at the uneven curves and edges of nature’s formations and the continuous gradations of colors found there. Another perspective is represented by Berlyne who believes that people often seek stimuli that can help them maintain optimal arousal levels, i.e., stimuli that have neither high nor low complexity [102,103]. Structures in nature are considered to have precisely such properties as they are often both uniform but at the same time varied [88,101,104,105]. As mentioned earlier in section Key Category 1, these structures that are ordered, uniform but at the same time rich in variation, can be described as fractal patterns, which are considered to facilitate the perceptual process according to the PFA theory [88]. Examples of such objects in nature that exhibit self-similarity on different scales, are clouds, snowflakes, and trees. When people look at pictures of nature’s fractal patterns, especially those with a medium degree of repetition and detail, or what can be mathematically described as a medium-high fractal dimension (D-value) between 1.3 and 1.5, EEG (electroencephalography) studies have registered activity in the mode of the brain associated with a wake and relaxed state, so-called alpha waves [106]. It is interpreted here as an opportunity to counteract stress by experiencing an inner state of calmness, which for children with autism could be a way to achieve increased emotional stability. Children with autism like to look for the predictable and therefore the way in which nature is structured, uniformly yet varied, is assumed to fit well into patterns and formations that they enjoy looking at.

Another concept that occurs in the literature on events in nature on the theme of unity and variety are movements that are constantly changing but at the same time remain the same, so called Heraclitan movements [107], and these movements are also associated with feelings of security and comfort. Here, the authors provide examples of watching fish swimming in an aquarium, grazing cows and horses, light and shadow play, or cracking clouds. As far as the children in the treatment are concerned, we have pointed out several different experiences in nature and with the animals that fit well with the theories and concepts raised here. However, the effects of riding have not yet been much commented on and the rhythm from sitting on the horse is also a form of recurring movements that the rider picks up via the body. The movements from the walking horse sometimes seem to have influenced the children to become more centered and vitalized afterwards. The children first looked satisfied and calm sitting on the horse, and then when dismounted, they again became more outgoing and alert, ready to engage in some activity. However, it is not always that riding had that effect which may have been due to the children’s state of mind when first arriving to therapy. Sometimes they were more talkative, showed joy, and were more outgoing direct during the riding episode, and there were also times when a child expressed a desire to walk instead of riding the horse. Whatever the reason, it is conceivable that the vitality forms triggered by riding the horse might have reinforced the emotional states the children had already from the start of the therapy, and perhaps also colored the children’s expressions and behavioral patterns, as the body signals may have been reinforced.

Many natural sounds also consist of repetition of sound patterns such as bird song, wind whistling in the trees, or rippling streams. Experimental studies have shown that people recover more quickly from stress after listening to natural sounds such as water from a fountain or bird chirps compared to listening to other types of sounds [29,108]. In other words, the natural environment in the treatment may have had continuous stress-reducing effects if the children now and then listened to the sounds of nature, which may have led to them being revitalized after a while with increased levels of alertness in accordance with Berlyne´s [102,103] and Wohlwill´s theories [101].

#### 3.2.2. The Stress-Reducing Effect of The Treatment Environment on Both Therapists and Children 

**Figure 5:**
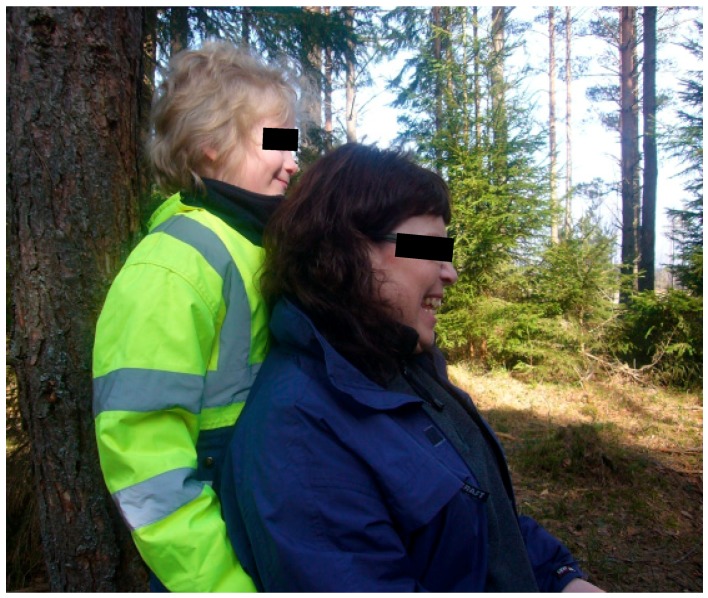
Soothing elements from therapy seemed to help the children to experience the surroundings as a safe place.

Nature has most likely also had stress-reducing effects on the therapists, which in turn may have reflected back on the children, see Figure 4. Although the treatment environment has largely been the same from time to time and therefore quite predictable, it has also been variable (seasonal changes, weather and wind, animal behavior variation, and variations in wildlife). Predictability along with changeability may have been helpful for the therapists to stay calm but still action-ready to be able to make an appropriate intervention. For example, the therapists could always be sure that there was something going on in the environment that could evoke interests in the children, they only needed to notice the right kind of moments and take advantage of them. For this to work, it was necessary to quite simultaneously read the child and what is going on in the environment. In other words, peace and quiet in connection with experiences from nature can take form in many ways. It may be about being able to view the environment from a protected site as in Figure 5 or being captured by a uniform but varied structure such as the fire in Figure 4.

#### 3.2.3. Summary Stress Reduction and Peace and Quiet Based on The Core Variable Vitality Forms

We believe that the elements of nature and animals in the treatment helped the children gain stress reduction. They also instilled a sense of calmness based on the more abundant and softer recurring movement patterns, visual and audial, that occurred in the natural phenomena and animal behavior. These patterns of movement and repetition of structures are thought to provide particularly beneficial forms of vitality that have stabilized the children emotionally, for example through a high degree of order and predictability. The children themselves seem to have managed to unconsciously tap into these kinds of experiences when they have been stressed, tired, unstimulated, or when they needed extended processing time. The vitality forms that these relatively simple visual or auditory patterns stimulated may also have contributed to revitalization in a way that did not lock their attention but rather seemed to facilitate shifts of focus to more dynamic events again. Children with autism often find it difficult to shift attention flexibly, especially when it comes to move focus by another person’s initiative. According to Stern, vitality can arise in a person not only based on physical movements but also based on mental movement for example by looking at an image or static stimuli [78]. Looking at nature’s formations, including fractal patterns, or listening to natural sounds can possibly be a kind of stimulus that can provide a good and tolerable arousal for a child with autism. By focusing on these more soothing specific vitality forms in nature, it seems as if the children have found a source that could improve their psychological balance in terms of access to psychological energy when needed. This might also enable them to occasionally be more active in exploratory play and interaction. Perhaps children with autism, who have an impaired ability to play using fantasy and pretense [22], can benefit much from such an improved energy balance, emotional stability and low levels of stress from vitalization coming from nature’s sounds, structures and formations.

### 3.3. Key Category 2. Awaken Curiosity and Interest

At all times, conversations about nature and animals have been used as an easy way to initiate contact between people. In fairy tales, fiction and poetry, the complexity of man is embodied by means of metaphors and symbols from the natural and animal realms. Toys in the form of animals are found in endless variations and the child’s so-called transitional objects (the link between the mother and the world) are often stuffed animals [109]. Wilson [110], in the biophilia theory, argues that, through evolution, humans have developed a special interest in animals and nature and that is why we so easily pay attention to them. He meant that man receives signals of well-being and security of seeing animals that are resting or relaxed. Kellert [111] describes biophilia as a result of biocultural development; it is an inherent tendency created through learning both culturally and experientially.

In a recent study on adults with epilepsy, a side finding was made that could substantiate the evolutionary aspect of biophilia. Individual neurons were found in the right part of the amygdala that responded strongly to the sight of animals, but not to humans [112]. As a reason, it was emphasized that animals played a major role both as prey and dangerous enemies during human development. The amygdala is a structure in the brain involved in detection of threats but also in the emotional evaluation and regulation of emotions [41], all of which are important functions in the area of mentalization. The survival of most animals is considered to be dependent on rapid detection and interpretation of other creatures’ movements, gaze direction, and social signals, according to Blakemore and Frith [113], who examined how the human brain manages to deal with the social world. They believe that the hypothesis of an evolutionary origin behind man’s more advanced social and communicative ability, e.g., being able to attribute mental states to other people, is based on simpler competence levels that we share with animals. An example of shared basic emotions between mammals can be the so called seven primary-processes of emotional systems provided by Panksepp [114]; seeking, rage, fear, lust, care, grief, and play. According to Blakemore and Frith however [113], the simpler competence level can be about perceiving basic emotions, gaze, biological movement, or goal-oriented action. If these authors are correct in their assumptions about a shared and basic way of being social and communicating among animals and humans based on rapid reading of biological movement, the step is not far to Stern’s thoughts [78] on the integrative functions of vitality forms in the mental towards holistic experience based on movement, force, space, time, and direction/intention. Perhaps children with autism have this basic tendency to be able to read animal behaviors based on movements and therefore have improved ability to engage in simpler social communication with them. The vitality that children with autism often show in relation to animals also sometimes seems to include humans. Several studies on children with autism and their interaction with animals report precisely increased social responsiveness as a noted effect of animal-assisted therapy [15,16,17,18,19,20,21]. We will cover biological movement a little further on in the text, but then in connection with the description of how some of the children in treatment move their bodies during certain types of games in nature. 

In research on the importance of nature for the development of children, it has been found that the elements and processes there can stimulate children’s interest, imagination, and emotions and help with associations, thinking, language, and to reflect [9,115,116]. This probably also applies to children with autism but with reservations that the stimulation does not automatically lead to a more analytical way of thinking such as understanding causal thinking, making comparisons, reflecting linguistically, or forming concepts. If exploration and play in nature takes place in a setting including interaction with other children, there is always also an increased risk of misinterpretation of behaviors that can easily lead to worry, fear, and to evasive behavior, or conflicts. However, it could be that with a long-term support from both therapists and the environment, as in the example here [68,69], the psychological development of the children with autism can be stimulated.

**Figure 6:**
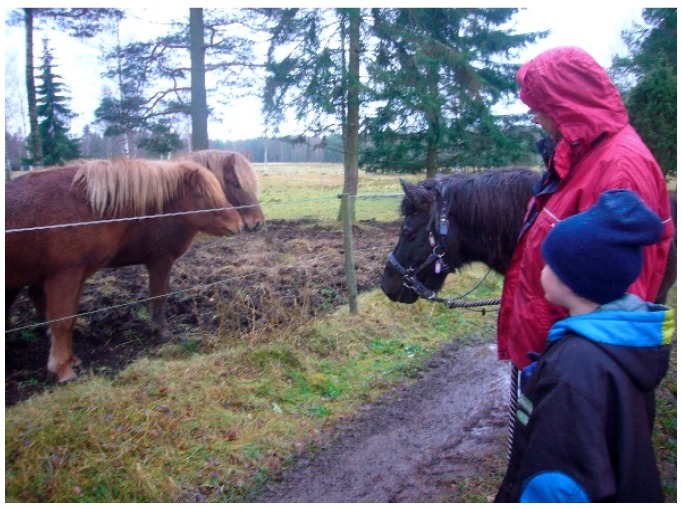
The horses in the pasture arouse curiosity and interest in the child which leads to shared moments and talk in a simpler dialogue with his therapist.

Figure 6 shows a typical situation for the connection between the child and the therapist in contact with one another based on the child’s interest in nature and animals. Below are also presented a more detailed example of a conversation from several therapy sessions. This because it clarifies the level of complexity in the child´s expressions and gives an example of how it gradually can become more coherent and well-connected to the therapist’s turns in the conversation, based on what is happening in the environment. The dialogue takes place during the walk to the forest on the boy’s first therapy sessions. The boy (B) has few words, his speech is unclear, and he is not easy to understand.

**First session**: B makes comments about animals he sees in the pastures as they pass during the walk up to the forest. The therapist perceives that he says: wants to cut the bangs; eat grass, we don’t.

**Second session:** B mimics all animals when walking. He thought it was fun to see all the horses in the pastures and he commented on this.

**Third session:** The therapist talks to B about horses in the pastures being curious. When they pass them, he says: They can´t see. The cow can´t see either! Then he said; Sara is happy (the dog) The handler then asks: Are you happy too? Then he says; you are nice!

**Fourth session:** Today B is talking more during the walk. He comments all the way up to the woods, he hums and the horses in the pasture neighs and the birds also chirped. The therapist then asks what B thinks they are saying. He replies: They think it’s fun that we come. Then he says hello to all the animals and hello to the forest and finally he adds: There our friends come! 

**Reflections:** The boy comments on the animals, compares humans and animals, he associates in thinking from animals back to himself (does not want to cut the bangs!). He takes the animal’s visual perspective, he comments on the dog’s emotional state, he responds with empathy to the handler’s question about how he feels and attributes her a positive trait, and when the therapist asks what he thinks the animals are saying to them, he manages to give an answer in a form of a more advanced explicit mentalization.

#### 3.3.1. Importance of Concrete and Clear Information from Nature Elements and from Animals to Stimulate Curiosity and Interest in Children with Autism

**Figure 7:**
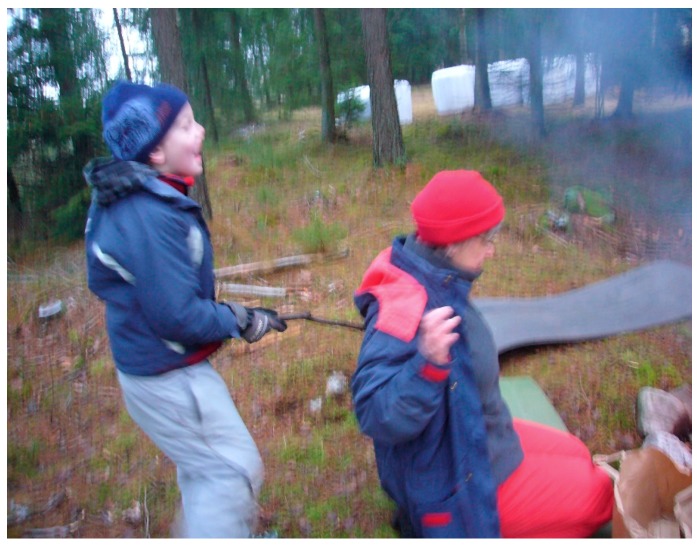
The boy pretends that the stick is a firearm and the handler plays along.

For children with autism, nature’s concrete multi-sensory stimulation is thought to be of particular importance since the group has difficulty in the more abstract representational thinking [117]. Gibson coined the concept of affordance about the awareness people have of their surroundings and its possible functions and meanings [118]. The concept has then been used in research about children and how children use nature in exploration and play. For example, a stick on the ground can be used as a firearm or as a tool to dig with, as in Figure 7. Kreutz [119] believes that nature with its varied possibilities of interpretations allows children of all ages and abilities to experience a certain level of what she calls "environmental congruence”. Nature’s affordances can be a form of sensory and concrete information that, with its clarity, helps children with autism become curious and show interest in what is happening and help them to imagine and form ideas about what they want to do or say. The direction of movements can, through registration of the vitality forms, provide information about intentions, and the goal of the movement, according to Stern [78]. It is conceivable that nature’s elements and animals’ behavior appear to be particularly easy to interpret for children with autism, both from the concept of affordances and the clarifying effect from registration of vitality forms involved. It is proposed that the vitality forms contribute to the child being vitalized. This vitalization thereby increases the child´s ability to get a clearer picture of the context and especially the animal’s forms of vitality often contribute to the formation of a small story. Stern [78] argues that all human actions, whether mental or physical, must adopt a vitality form as they unfold and that we do not notice this because the forms of vitality fuse with thoughts. Stern expresses it as follows, “They are absorbed into the explicit meaning as the vitality form accompanies a train of thought” ([78], p.10). What he proposes is that the vitality forms are of great importance for thinking, feelings and sensations to be experienced as dynamic and whole experiences, even though the impressions come from different modes. Children with autism are likely to be helped by this type of dynamic experiencing when it comes to helping them to understand and interpret what is happening. A more global thinking based on the brains ability to integrate impressions from many sources, i.e., central coherence, is one of the main difficulties in autism [39,40]. If the children with autism seem to experience vitality directly from social interaction and communication with another person about what is happening in the moments of events during therapy, there may be a reason to assume that it is precisely the forms of vitality that contribute to this. The children have often showed social interest in talking about what is happening, something that pictures 11–14 can show examples of.

**Figure 8:**
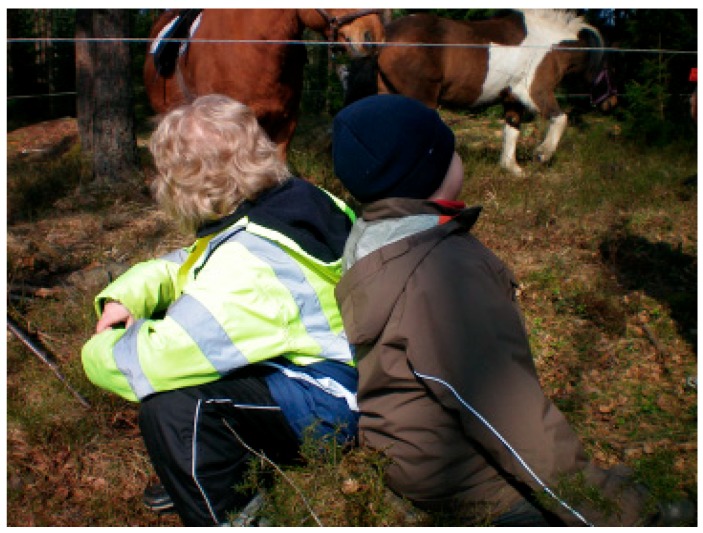
Two boys chose to sit close to one another both looking at the horses—perhaps mutually shared moment of contact between them?

As we have previously touched upon, animals can also be a form of affordance for a child with autism in that they offer social interaction on a simpler basis than interaction with humans, as can be seen in Figure 8. Here, it is suggested that both the concept of vitality forms and the concept of affordance go hand in hand in providing contextual meaning to children with autism.

Some of the children seem to be quite good at reading other children’s and adults’ rapid movements in games such as chasing one another. The question is whether this type of game is about a simpler form of coordinated action with old roots. Animals often move in a way that clearly show their intentions as they do not mask their behavior, which can mean that they convey their vitality particularly well. With a little practice, several of the children have seemed to be able to adjust to some extent how they move in relation to other partners in the game of chasing, where both parties must read intentions through body language and glances. The game reminds of a kind of hunting scene and has probably been played by children of all times, even when mankind was hunters and gatherers. The vitality forms that accompany this type of social interaction may also have helped the children to better integrate and understand what was communicated, both verbal and nonverbal, provided that they were focused and interested. If the mental content is enclosed by the so-called vitality representations, as Stern describes they do [78], this in itself may also have affected the encoding of the experiences in the episodic memory, leaving independent tracks for recalling, alongside the so-called content representations. Some especially good photos in relation to positive emotions and radiation of vitality from the children’s actions, were sent home after each session. This with the intention of positively influence the children’s growing functions by helping them to recall and retell valuable memories, for instance in conversations with their parents or other people outside the family. 

The therapists have noticed that the interest the children in therapy initially showed for the events in nature and animals also persisted throughout the treatment. This interest grows more and more to include even the therapist’s words and responses in the communication which gave way for them to support the children in understanding more complex situations. The conditions for children’s meaning-making in exploration and conversation have probably improved by this and equipped the children to become more and more engaged in social interaction, both with animals and people with perhaps even more vitality as a result. Learning has thus been bodily experience-based, where both vitality forms and emotions evoked have evolved to become important amplifiers. In this way, nature and animals integrated with the therapists’ interventions have become a way for the children to reach a certain level of what Kreutz describes as environmental congruence, i.e., a good match between the children’s need to develop their functions further and what the treatment environment has to offer [119]. For children with autism, it is not easy to spontaneously share joyful experiences with other people, something that has often happened in the treatment.

**Figure 9:**
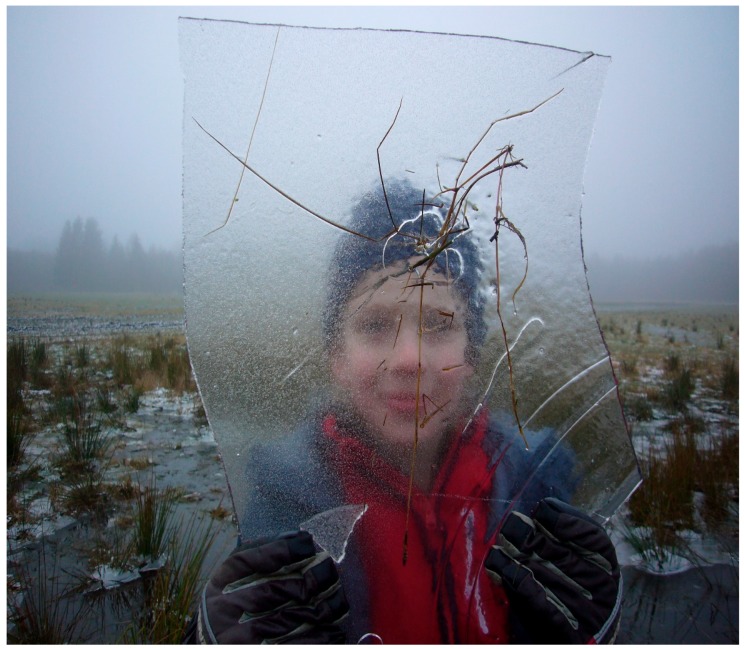
The boy picked up a piece of ice and held it in front of his face. He then asked the therapist to take his photo. Why did he do this? Perhaps he felt that the ice gave him a comfortable shelter in exchange of gazes, or he was simply curious what he would look like or he wanted to tell something about himself that could lead to confirmation by the therapist? We will never know for certain, but the fact is that the boy acted creatively with the help of one of nature’s formations and he very much liked to look at the picture afterwards.

In slightly more developed children in language and cognition, we have noticed that processing of more psychological content could be initiated based on the children’s interest in nature’s more concrete and sensory stimulation. Among various elements of nature, cones, stones, and ice have been specifically used by the children engaging in these more symbolic acts of play, see Figure 9. The question is whether the symbolic language based on the formations of nature that the children have perceived visually has helped them to process thoughts and feelings? Children with autism find it difficult to use imaginary thinking [22], and the language is often stereotyped and concrete without the use of metaphors or imagery.

#### 3.3.2. Sensory Processing and Mental Processes Based on Experiences of Nature and Animals

Natural environments can have specific advantages over indoor environments when it comes to processing special visual and auditory sensory information [86,87,88,108,120,121,122]. This could be particularly valuable effects for younger children with autism, as they have difficulty understanding and linking their experiences with language, which impairs their sense of overall understanding what is going on. In general, the visual sense channel is usually the strongest in autism. Kana et al. [123] have shown that for a group of adolescents with autism included in their study, visualization was an important strategy for understanding read sentences. The study supported research that has shown that there is an under integration of languages and imagery in autism [124,125] and the authors concluded that people with autism are more dependent on visualization, as a support for language understanding. Many of the cognitive tools that have been developed to facilitate learning in autism are just visual in nature such as, for example, pictures, photos, and schematics. In nature, the experiences, just as in any other everyday environment, are primarily visual (but also multi-sensory) which can facilitate the children to process and understand what is happening and communicated, both nonverbally and verbally. The fact that children with autism often rely heavily on the visual channel for learning may have to do with the fact that vitality forms from visual impressions for them (not regarding social signals from human communication) could be particularly easy to register. In that case, contextual information could be more efficiently provided and better understood. One can assume that the visual aspect of capturing biological movement is also particularly important.

Another sensory aspect of the natural environment that can facilitate understanding of spoken language is the sound based aspect. An example is Christensson’s research [122] which shows that sound perception in the form of listening to another person’s speech is improved in a forest environment since the sound reflexes there are different. The consonants are emphasized and not the vowels (as in many indoor environments), and that makes the meaning of speech more effective, according to Christensson [122,126]. This can be important for understanding why the children’s interest in nature and animals in treatment could be maintained, and it could be partly from listening and take in the words from the therapists explaining. Interest in animals and nature as objects of conversation can also, with some simplicity, be directed towards social domains which is something very valuable for children with autism since they do not readily absorb or are interested in human social knowledge mediated directly from social interaction with people. In other words, there may be several advantages from reading stimuli coming from the natural environment and animals in treatment that can improve the children’s ability to understand and communicate verbally.

Another important aspect based on vitality forms from stimuli in nature could be that in nature there are so many different categories of multisensory events that humans through evolution could have developed a functional way to tap into, understand, and take advantage from, without consciously being aware of it. Other theories such as SRT [86] and PFA [87,88], both put emphasis on quick or effortless perceptual processes, and perhaps vitality forms have a part in this. Intuitively and flexibly we could then either turn our attention for a moment to an event that gives us energy through the specific forms of vitality we need for the moment, or we let objects or events capture our attention spontaneously and then we can choose to explore it more closely if a more intense interest is evoked. Both in the next key category and in the discussions, shifting of attention based on vitality forms will be further discussed.

#### 3.3.3. Different Emotional Reactions Based on Curiosity and Interest in Nature and Animals

**Figure 10:**
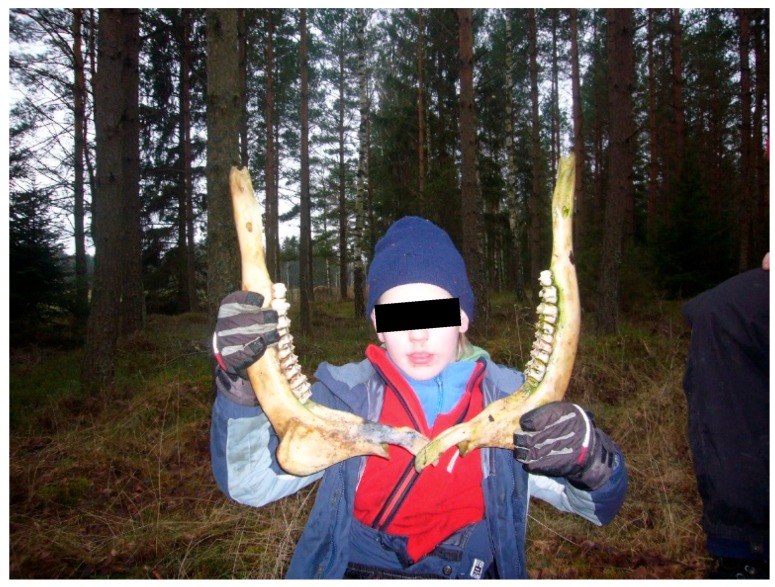
Going for a treasure hunt in the forest was something that all the children liked, and aroused their curiosity and interest, which stimulated thinking and language.

Children’s development always includes experiences of both positive, neutral, and negative colored emotions. The emotions aroused from inputs of treatment have usually been positive, but negative emotions such as fears have also occurred, but not to the extent that they could not be overcome with the help of the therapists’ interventions. For the most part, however, worry and fear have originated from social interaction between the children. Objects in nature or animals have seldom aroused fear, but there have been such events too. For example, one child was afraid to ride and has only mounted the horse one or two times. Another child gradually started to like horse-riding, and much liked it thereafter. Yet another child was fascinated, but at the same time fear-filled, when looking and coming into closer contact with ants and insects. This interest followed him for several sessions, where he eventually mastered his fears to the extent that he could hold out a stick of creeping ants to other children and calmly asked if they feared them. The incident with the ants seemed to be a kind of challenging experience that helped the boy reach a higher level of mastery in regulating his behavior. The fact that he himself could control and independently choose what he wanted to do, seemed to have strengthen him as he looked very happy and satisfied. In other words, nature can give rise to many kinds of experiences, including the biophobic fears of spiders, snakes, stagnant water, and dark caves. These biophobic responses, as well as the biophilic ones, Ulrich considers to have genetic basis as both are adaptive responses that have been important to learn and retain for survival [100]. The so-called negative reactions, if moderate, can, with the help of the therapists lead the child to a stronger self-reliance and therefore become very important experiences of mastering. The deeper forest on the outskirts of the environment has inspired to make more danger colored fantasies, but not in too overwhelming ways. Sometimes these fantasies have seemed to reinforce a sense of belonging in the group as they were narrated and shared between the children and their therapists. For example, fantasies could be triggered from traces of wild animals or skeletal parts the children found, see Figure 10. For children with autism, it is particularly important to experience what it is like to get help in dealing with situations that cause anxiety or fear, for example by turning to an adult or as here to a therapist or see how other children and adults solve problems they encounter. Tomkins, who developed the modern theory of affect [127,128], describes affect as a separate but reinforcing coordinator in our psyche that can flexibly serve as links to other basic systems such as drift, motor, cognition, and perception. A thought that follows an affect can be reinforced and become part of a script. A script according to Tomkins is the organizing principles used to interpret, respond to, and control experienced episodes, so-called scenes. According to Tomkins, the meta message from positive affects such as joy and interest signals *continue* (the search for novelty is a strong motivational factor in infants) and the negative ones (anger, fear, sadness, disgust, and shame) signal *change something*. The children in the treatment have received a good deal of stimulation from nature and the animals because they have become sufficiently curious and interested in what has taken place there. In this way, they have been supplied with mental energy and vitality continuously.

During one of the walks, a skeleton was found of a moose jaw and in connection with this event, a boy (not pictured) starts talking more intensely and fluently with his handler about dangerous things like trolls, bombs, and spiders, but also about elephants and dinosaurs. He describes a fight that he himself wins and he starts counting horses in the pastures, something he continues to do on several following sessions. He also remembers the incident at the next therapy session a week later and suddenly starts to talk about dinosaurs and wonder if they are there. The boy also begins to tell other children in his simplified way about the wild animals in the forest; lists different animals that lay eggs*... and then kids come* (the animals’ kids)*, do not hurt ants* (when a boy is looking for insects with stick in the ground). He also tells the therapist that he thinks some of the horses are very nice. When he finds additional skeletal parts, he fetches the therapist to show her. The stimuli in nature that evokes moderate fear had a particularly important motivating force and aroused a lot of interest and curiosity in the children that inspired their thinking processes and could lead a train of thoughts to be articulated. The vitality of the children in these situations has been particularly obvious and forceful.

##### Summary Arouse Curiosity and Interest Based on The Core Variable Vitality Forms

In the treatment, children are often revitalized by all the dynamic experiences they are involved in with nature and animals and the prerequisite for this can be the vitality that has arisen from a first curious and interested look at, for example, a bird that has flown up or an ice cube trampled on or a stick on the ground. An inherent interest for lifelike processes, biophilia, (although the hypothesis is not yet fully confirmed by the research) may possibly be intact in children with autism and thus form a way for therapists to get children with autism to become increasingly more socially interested, more willing to show and comment and more frequently wanting to share their experiences spontaneously with others. If the children are socially engaged and experience joy and other positive feelings in communicating with another individual, there is also a possibility that this in itself can become vitalizing experiences that produce increased mental energy rather than reduced.

We hereby propose that vitality forms from nature and animals experiences in treatment, could have been more effectively registered than earlier research has shown to be true for children with autism in relation to human movement patterns [78,84]. With such improved encoding of vitality representations, children can more easily recall their memories and talk about them. If this is a correct hypothesis, the question is whether this also improve conditions for the formation of the so-called. RIGS (Representation of an Interaction that has become Generalized) according to Stern’s theories [80]. With RIGS, Stern refers to the way in which young children’s real experiences of interaction are organized in the psyche [80]. Stern links the emergence of RIGS to memory structures that represent perceived experiences. These models of “being with others” children can start to recall from an early age in structures similar to stories. According to Stern, such a story contains different parts, for example, sensory impressions, actions, effects, and goals and they form a temporal and thematic coherent unit. These experiences of interaction can then be generalized to form RIGS. An example might be when the baby interacts with the mother when playing peekaboo. Here, instead, the experiences of interaction can be about how to play hide and seek with others, how to hold the cat or pet the dog, how to interact with the handler when resting, or how to ask when you do not know or express your own needs so that one could be understood, all experiences originated from being socially engaged and interested in what is happening around you. Possibly RIGS formed during treatment then also could guide the child even in other contexts. 

### 3.4. Key Category 3—Use of S Attention Spontaneously in Exploration, Play and Interaction

Attention restoration theory (ART) is based on the assumption that people have two systems of attention [120], directed attention (first referred to as voluntary attention by James [129]) and spontaneous attention also known as fascination (first called involuntary attention by James [129]). Fascination is an object or event that catches our attention and which we do not need or can decide on. Directed attention is used when we work in a targeted manner and must inhibit distractors, which requires a lot of energy and is an exhaustive resource involved in executive processes in thinking. Nature is a place that easily arouses soft fascination [120] (for example, by moving objects, objects that are particularly beautiful, or by wildlife). In studies, nature has proven to be a good place for recovery after directed attention by stimulating soft fascination [120]. This effect is particularly significant in many of the practical applications of nature in treatment that exists today [1]. One example is horticultural therapy, a well-regarded adult treatment for conditions such as exhaustion disorder, depression, and stroke [130], all of which are characterized by severely impaired executive function. With the help of the soft fascination we use in nature, we can quickly and unconsciously decode the environment’s both soft and hard elements of fascination, says Ottosson et al. [89]. With objects that attract our attention with hard fascination (e.g., watching exciting sports events or being close to high precipices or big snakes), Kaplan [120] believes that all our attention is required, and we must be vigilant which leaves no room for our own thoughts or reflections which soft fascination does. Kaplan shows the possibility that nature is a place that can easily arouse interest and thus the use of soft fascination.

There is also research showing that outdoor environments have salutogenic effects on children’s attention [131,132]. In a study involving children with attention problems, parents reported a reduction in symptoms (ADHD) following play in green areas [133]. The kind of dysfunction within the executive function of children with autism, primarily is about difficulties in shifting attention and multitasking [37,38]. According to Kaplan [120], directed attention is used in virtually all executive functions, including also to shift focus and to handle more complex patterns of action. However, it is important to remember that in all young children, the executive function is undeveloped from the start in life and therefore the support from the caregivers are especially crucial during the first years of life [134,135]. In the light of this, the executive function matures with age and the child becomes increasingly more able to independently make plans and organize his thoughts and behaviors. In other words, children need a lot of appropriate confirmation and support from adults in their environment to develop their executive function, something that is not so easy to achieve for children with autism. This is why nature and animals are especially important objects as they attract the children’s interest spontaneously which gives the therapist a chance, when needed, to fill in words and cues when they still have an open mind and seems to want help to organize the information that could improve the understanding further.

**Figure 11:**
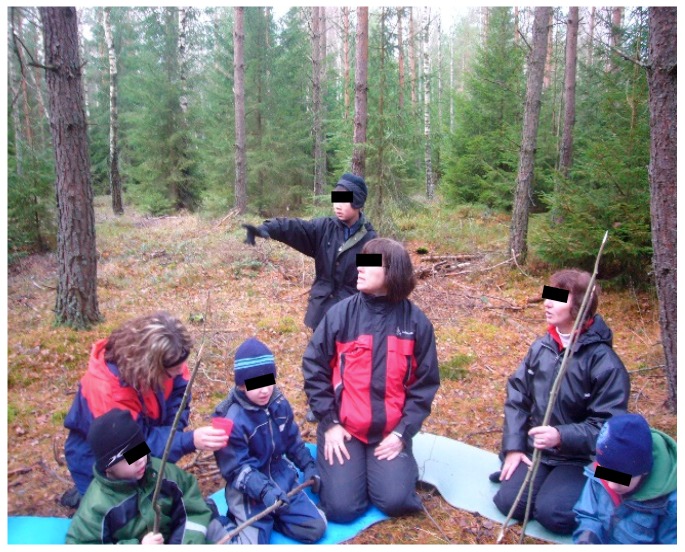
The child looks and points to show and share an experience in nature with therapists. Children with autism seldom point to show and share experiences. The question is whether the use of spontaneous attention also positively affects the use of body language?

**Figure 12:**
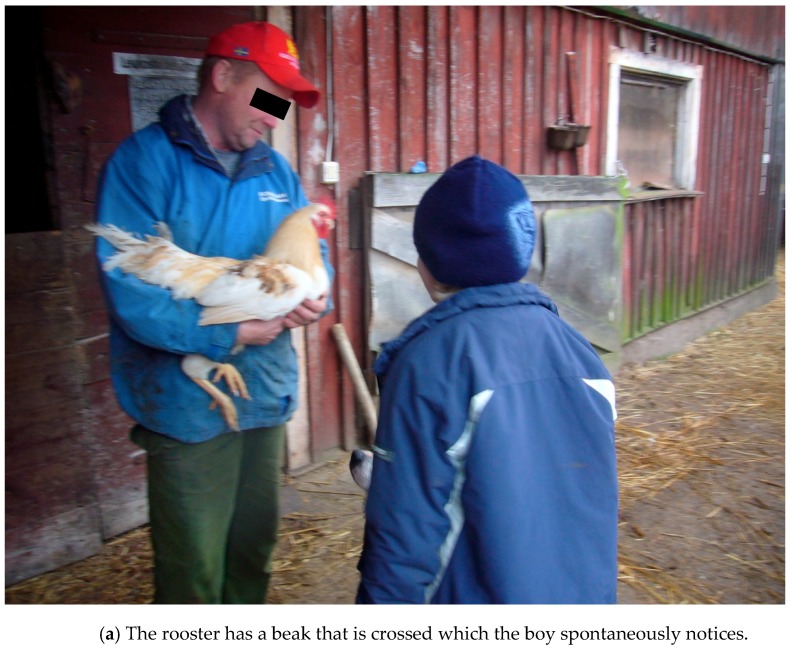

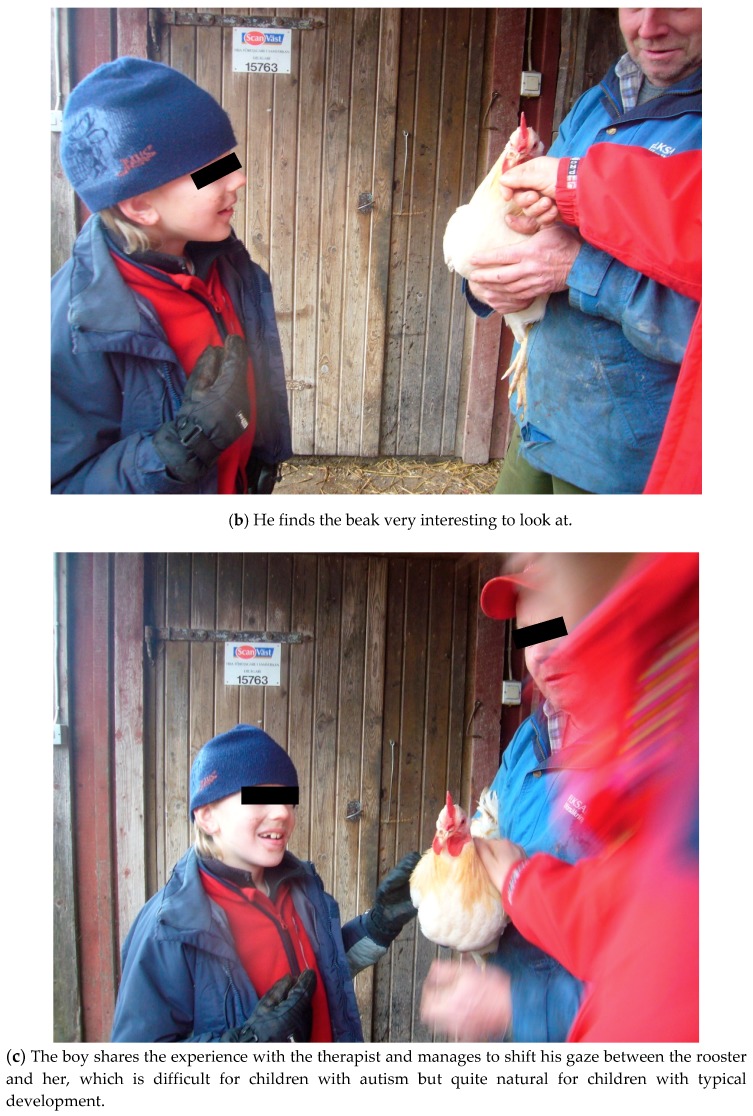
The boy finds the rooster’s crossed beak very interesting to look at and shares his experience with the therapist in a joint attention episode.

The treatment referred here, differs from traditional treatment for children with autism in that the children participate to a greater extent based on their own initiatives and spontaneous engagement. The farm and surrounding nature are not as structured environment as is otherwise characteristic of educational environments for children with autism, but still appear to be supportive of the executive function. One way to indicate this is that the therapists do not have to lead the child as much, but the children themselves seems to be able to keep ideas about how the environment is structured. This can make the children freer to choose activities, which probably strengthens their motivation and the sense of self-managing their actions (agency). In many forms of treatment for children with autism, the therapist leads the child and has much control. An important task for the therapist in dealing with animals and nature in our example, is to listen carefully, be perceptive and responsive to the child’s needs. It is also important for them to actively monitor and share what the child spontaneously notices or could be interested in, and to put words on experiences when appropriate and with good timing. The impact of the events on the children’s attention, is probably a crucial circumstance for this to happen, as is shown in Figure 11 and Figure 12a–c. The executive function of the children seems to be either relieved or supported continuously by both the therapists’ interventions and by the environment and probably the vitality forms from all the dynamic events has something to do with this. According to Posner [135], an alert state is necessary for more demanding thought activities. It is striking to see how the children in the treatment can go from a resting state, e.g., lie down or sitting on the ground looking around, to getting up and being active in games like hide and seek with just a little help from the therapist or sometimes no one at all. In other words, the ability to shift attention seems to be reinforced, something that we also commented on in both key category 1 and 2. In the second project included here [69], a different test for assessing mentalization (first order false-belief test) was used in the pre- and post-evaluation with clear positive results for three of four children with autism (the fourth was due to dropout). In addition to mentalizing, the tests include demands on executive function such as working memory and inhibition.

In the following section, a girl’s more advanced ability to reflect verbally, is described when she spontaneously draws the attention of her therapist to a situation with a cat she is interacting with. The background story is that a cat often approaches the children when they are sitting at the campfire and he always wants to have food and attention from them. At the beginning of the treatment the girl had great difficulties with impulse control which the therapist helped her to improve. One time at the end of the treatment, she wanted to show her therapist how good contact with the cat she developed. She says: *Look, when I release the cat it comes back to me!* (and exactly this happens) Then she adds: *Wonder if the cat owner knows who tamed it?*

**Reflections:** These are examples of a growing mentalizing capacity and shows the girl’s reflections on how another person can think about her indirectly, through the cat’s behavior. This type of reflection is unusual for a child with autism. Similarly, it is unusual for a child with autism to spontaneously call on someone else’s attention to show and share joy and pride from own progress. This is not an isolated story; all the participating children have shown a more elaborated mentalizing ability to reflect in conversation after some time in therapy. Not all at the same high level as this child, though.

**Figure 13:**
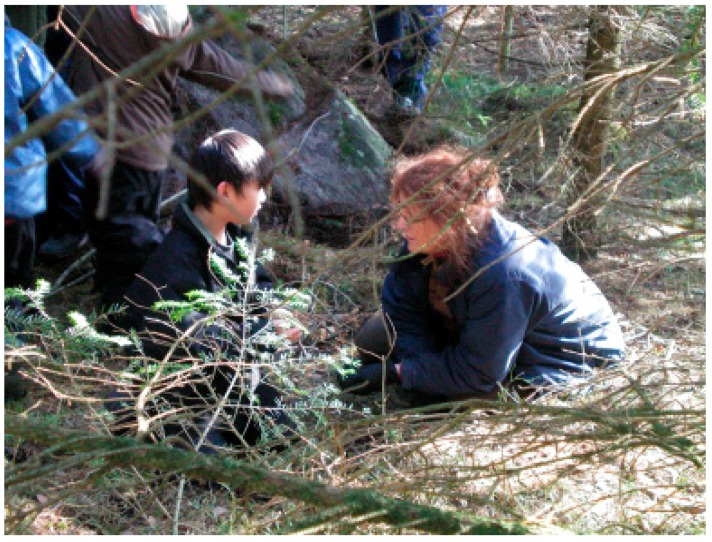
In-depth contact during the pretend play based on spontaneous attention for other children’s games in nature.

Other children’s pretend play may also trigger pretend play in children who are about to develop this form of thinking. An example comes from an episode that took place towards the end of the first treatment period when a boy was included in the other children’s more advanced pretend play on a big stone, see Figure 13. They pretended that they were fishing for whales in the Arctic. The game was too difficult for the boy to understand but suddenly he stood there and watched them. After a while he pulled out a small wilted fir and said something that could not be heard. The therapist replied... good, you have a fishing net... and he answered... yes! When she cooked soup with the fish caught in the net, the boy contributed with roots and other things he fetched but wondered at first; ... is it not soup? Well, we pretend it is, the therapist then replied. The boy showed great interest in the pretense and was very focused. Some weeks later it was time for a school follow-up. When we asked if he was playing in the school yard, they answered that he had just started a kind of pretense play about cooking soup with another girl in the class. He had clearly transferred his new skill to another environment, something unusual for a child with autism to do, especially if the skill is not part of the child´s special interest.

#### 3.4.1. Soft Fascination in Unexpected Events

A particularly important quality of treatment that has attracted much of the children’s attention spontaneously is when something happened suddenly and was not expected. For the most part, the events in therapy took place in a varied, yet relatively uniform manner. In nature and in animals, for example, there is always some form of movement activity in what is going on. Trees and foliage move in the wind, the animals walk slowly grazing in the pastures. Sometimes this relative uniformity is broken and something new happens, like when a bird suddenly flies up from a branch or when a horse neighs and curiously approaches the fence in the pasture more eagerly than otherwise. These more divergent episodes in nature have been used by the therapists in their interventions to explain something. They also may have formed a starting point of a more playful conversation that raised the mood, and this sometimes helped the children take new steps to use their imagination. An example of this was when one of the farm animals suddenly showed more amused and cheerful behavior than they otherwise used to do. The piglets for instance aroused much joy and laughter when the children watched them play and jump in the straw. These experiences gained even more value when narrated in the group at the campfire. In other words, sudden events added more powerful dynamics to the children’s experiences and formed almost a kind of accents that seemed to give them more mental energy and vitality and attracted their attention in a more powerful way than from calm and recurring softer movements. The forms of vitality involved may have strongly colored the children´s emotions and so influenced how the final expression were perceived. Children with autism often have difficulties with unexpected events and may perceive them as disruptive elements that rather prevent their mental processing from being flowing, and efficient. By managing to keep their attention a little longer for what is new and little unexpected, the chance increases for the therapist to reach the child with additional structuring support. In this way, the somewhat unexpected events have sometimes become decisive moments, which gave the children new perspectives.

#### 3.4.2. Summary, Spontaneous Attention Based on The Core Variable Vitality Forms

**Figure 14:**
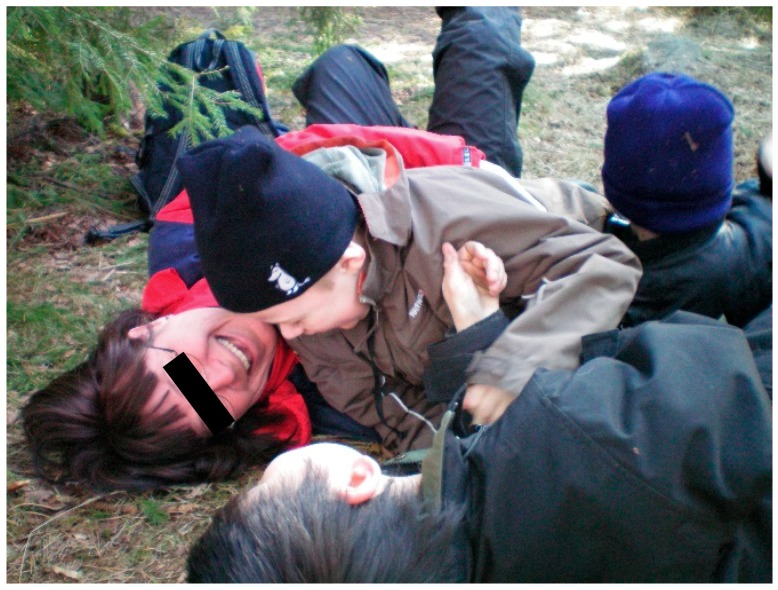
Most children appreciated physical play. The therapist helps to signal boundaries.

Here we discuss the possibility that events in nature and from encounters with the animals during the treatment, sometimes led to more dynamic vitalizing experiences for the children, as a result from their attention being attracted spontaneously. Such attention to events and objects can also mean more of their own spontaneous body movements, increased vitality, and more positive expectations about what will come next in the moments to follow. This kind of vitality dynamics may have provided the children with extra mental energy on certain occasions, which could have facilitated increasingly more complex thinking processes, for example executive function or mentalization. In treatment, physical interaction and games have been very dynamic (e.g., hide and seek, throwing snowballs, or playing with the dog) which required some ability to read other people’s nonverbal and verbal communication. When the children chased one another, it was required of them both to look at how the other children moved and at the same time monitor their own movements, thus be able to run away or to catch the other. Figure 14 shows two boys involved in a game that was about falling to the ground on a given time signal.

The increased vitality and alertness from all the movement dynamics may also have increased the children’s capacity to shift attention more flexibly, control behaviors, such as going from activity to rest, or during a moment be able to take part in more demanding social interactional episodes. Nature and the animals’ more unregulated course of events, with room for random and, to some extent, unexpected events, have been particularly important moments that the therapist could use to achieve more vividness, wonder, and joy in the mutual contact and interaction with the child. This may also have increased the children’s openness and ability to register the forms of vitality even from the therapist’s (or other participants in the group) communication and body language. Even the rhythmic stimulation from the riding may have constituted such vitalizing dynamic experiences (apart from the calming experiences reported on earlier) but in a different way as the child in that position must accept to follow the horse’s movement pattern. The child can, however, influence the horse’s movement to some degree by sending different body or voice signals, getting the horse to walk, stop, etc., however, they need to check back with the handler. Horseback riding then seems to evoke a specific kind of dynamic vitality from the communication with the horse and the handler based on a more tight form of joint attention situation which also could mean increased use of the directed attention from time to time, for example when to listen attentively and follow instructions. In playing games in nature, the child can move the whole body quicker and freer according to their own will, needs, and desires, and also to some extent, in accordance with how other partners in play move and show their intentions. This seems to involve more of spontaneous and quick switching of attention and checkbacks in joint attention with other people. Both situations are thought to be significant as good quality experiences of social communication acts. The free play situation though seems to invoke more dynamic flow of vitality than the horse riding, but both contribute to the child’s growing experience of being in tune with other living beings in a way they appreciate and often are seen to take much pleasure from.

## 4. Discussion

The purpose of this article was to seek a theoretical model that could provide increased understanding of how nature and animals can bring positive values for the psychological development of children with autism, in the context of a developmental supportive treatment. It is suggested that it might be through an improved ability to register vitality forms from all the experiences that the children were engaged in. More effective vitality form registration can have improved other cognitive and affective functions, for example the ability to grasp whole events, the so-called central coherence. We also suggest that the vitality forms have explanatory value for most of the examples presented in the three different key categories. The first key category describes how nature and animals appear to help the children reduce stress and provide calmness through all soft and repetitive recurrent movements that occur in abundance in nature and from animal behavior. The second key category addresses the fact that the children’s curiosity and interest are stimulated by nature and animals, which vitalize their thinking. The therapists used this heightened interest for their interventions, for example to coordinate the child’s attention with their own in so-called joint attention episodes or to provide rich linguistic stimulation and support. The third and final key category describes that nature and animals have attracted the children to pay attention spontaneously. This seems to have provided them with a higher degree of vitality and mental energy from all the movement dynamics involved. The resulting alertness in the children, might explain their increased ability to reflect in conversation, engage socially, and use more fantasy in play. The spontaneous acting and engagement also seemed to have improved the children’s capacity to engage in a more affective based communication style when communicating with therapists and other participants. This is something that children with autism find difficult to do, which hinders them from grasping other people’s minds [49,136]. By processing social information in conjunction with nature and animals, other people’s emotions can perhaps become more reachable to them through mirroring. If this conclusion is correct, more optimal conditions for psychological development could come out of this interaction with nature for young children with autism. Perhaps many children with autism can have their psychological development improved in the same way as the children included in this study. Especially if time in treatment is long enough for schemas, inner representations, and the so called implicit relational knowing referred to by Stern and his colleagues [80,137] to be established and stored in memory.

However, the study is based on limited empirical material. The theoretical model needs to be developed and revised through knowledge from a larger sample, including controls and if possible, randomization. Here the children have acted as their own controls. The researchers should also not be the same persons as those involved in the actual therapeutic work, as was the case here, where the first author has been involved both in the research and model building as well as in the practical therapy on which the result is built. A fact that minimizes a risk for conformational bias, is that the conclusions about the children’s progress in therapy build on many sources, and there has been a lot of transparency and discussions and feedback between all parties involved, therapists, teachers, and parents. 

If vitality form registration in connection with animal and nature experiences are a key for children with autism to become more receptive to social signals from humans, or to be able to process information in general in a more optimal way, then several new questions arise. For instance, which dynamic movement patterns could be the most optimal for this to happen? Probably it is important that vitality and mental energy is derived from being both mentally and bodily active but in a way that makes sense to the child. That way, creativity in thinking and openness to new experiences might be triggered. An advantage with a more relational based treatment as the one used here, is that the therapist works intuitively and can adjust to each child’s needs from moment to moment. The therapeutic environment is also quite wide and varied which can mean that it might suit many children with autism, but of course not all. Some children can have sensibilities and ways of handling information that mean they need a more structured and controlled environment for learning. Also, there might be children with autism that do not respond well because they lack interest in animals, are too afraid of them, or for some other reason do not grasp the meaning coming from nature, or are not ready to engage socially. In the treatment included here, many different sources of dynamic experiences coming from nature and animals have been used. We think this has been very important in order to create many varied opportunities for the children and their therapists to engage emotionally and socially. By following up and give affirmative support directly linked to the children’s initiatives and actions, conditions for development are thought to have been particularly optimized. With more inputs to reflect upon mutually in conversations, the more opportunities arise for the therapist to give the child abundant and nuanced affirmative support. This could mean increased possibilities for the children to receive and incorporate just the kind of experiences they need for their new emerging functions to develop further and for a sense of self to grow.

All in all, we suggest that the use of directed attention, the more energy consuming form of attention, is less demanding if the interactional activities with other humans take place in a nature-and animal-based context than otherwise. This could perhaps lead to improved attentional capacity in other settings where the directed attention is more needed. Further research could address this question. Participating in therapy like the one we have presented here, might also provide good conditions for maturation of executive function and mentalizing capabilities. Today, many children and adolescents with autism experience stress, and struggle much to achieve their educational goals in school, and many still need a lot of help for their learning and functioning, despite the fact that they during preschool time received a lot of intensive training [138]. Perhaps, beside certain typical autistic dysfunctions, they also lack a sense of self agency and self-control that children with typical development gain from their social and emotional early experiences. It is therefore very important to find support and social arenas where children and adolescents with autism can feel safe, motivated and able to use a more playful and spontaneous attitude in activities and at the same time to some extent, be socially engaged. If so, vitality might provide them with mental energy enough to overcome some of the obstacles in functioning and for being more directly connected to both humans and animals.

## 5. Conclusions

For thousands of years, humans have been adapted to interpret the behavior of animals and events in nature [80,82,113] and according to Hofer [133] this may also have been related to vitality forms. Children with autism do not seem to register or are sensitive to vitality forms coming from other people [78] which probably impacts their social understanding to a large extent. The question raised here is if children with autism could build relational based knowledge more effectively if the social engagement with other people takes place in nature and in conjunction with animals? If this is true, it could mean that children with autism can use a way of thinking that to a lesser extent is influenced negatively by autistic thinking, by using older systems to process information from the environment, discarded in man as a result of evolution. 

Perhaps in the future a more relational based treatment in nature and with animals can become a comparable alternative to other interventions being best practice today. Either way, it seems likely that animals and nature in the future will be part of a new avenue of treatments to instill psychological development and wellbeing for people with autism, young and old, just as research has shown to be true for groups such as elderly people, people suffering from stress and mental fatigue, or other conditions affecting the executive function negatively.
